# A framework for streamlining research workflow in neuroscience and psychology

**DOI:** 10.3389/fninf.2013.00052

**Published:** 2014-01-17

**Authors:** Jonas Kubilius

**Affiliations:** Laboratories of Biological and Experimental Psychology, Faculty of Psychology and Educational Sciences, KU LeuvenLeuven, Belgium

**Keywords:** python, neuroscience, vision, psychophysics, fMRI, MVPA, reproducibility, collaboration

## Abstract

Successful accumulation of knowledge is critically dependent on the ability to verify and replicate every part of scientific conduct. However, such principles are difficult to enact when researchers continue to resort on *ad-hoc* workflows and with poorly maintained code base. In this paper I examine the needs of neuroscience and psychology community, and introduce *psychopy_ext*, a unifying framework that seamlessly integrates popular experiment building, analysis and manuscript preparation tools by choosing reasonable defaults and implementing relatively rigid patterns of workflow. This structure allows for automation of multiple tasks, such as generated user interfaces, unit testing, control analyses of stimuli, single-command access to descriptive statistics, and publication quality plotting. Taken together, *psychopy_ext* opens an exciting possibility for a faster, more robust code development and collaboration for researchers.

## Introduction

In recent years, Python and its scientific packages emerged as a promising platform for researchers in neuroscience and psychology, including *PsychoPy* for running experiments (Peirce, 2007, 2009), *pandas*^1^ and *statsmodels*^2^ for data analysis, *PyMVPA* (Hanke et al., 2009) and *scikit-learn* (Pedregosa et al., 2011) for machine learning data analyses, and *NeuroDebian* (Halchenko and Hanke, 2012) as an overarching platform providing an easy deployment of these tools. Together, these tools are increasingly opening possibilities for development, sharing and building upon experimental and analysis code.

However, with most software focusing on facilitation of the various parts of scientific routine, up till very recently there were few if any options to directly foster the key principles of science, namely, transparency and reproducibility. Even with an increasing interest in Open Science, it is very infrequent that a researcher publishes the entire log of her work that would allow for a perfect reproduction of each and every step of that work. In fact, while open access to publications is largely perceived as desired, open sourcing experiment and analysis code is often ignored or met with a grain of skepticism, and for a good reason: many publications would be difficult to reproduce from start to end given typically poor coding skills, lack of version control habits, and the prevalence of manual implementation of many tasks (such as statistical analyses or plotting) in neuroscience and psychology (Ince et al., 2012). As many practicing scientists know, organizing different research stages together into a clean working copy is a time-consuming and thankless job in the publish-or-perish merit system. Yet these tendencies are troubling because lacking software engineering skills, researchers are more likely to produce poor quality code, and in the absence of code sharing, errors are hard to detect (Joppa et al., 2013), leading to reproducible research in theory but not in practice.

I argue that the primary reason of such irreproducible research is the lack of tools that would seamlessly enact good coding and sharing standards. Here I examine the needs of neuroscience and psychology community and develop a framework tailored to address these needs. To implement these ideas, I introduce a Python package called *psychopy_ext* (http://psychopy_ext.klab.lt) that ties together existing Python packages for project organization, creation of experiments, behavioral, functional magnetic resonance imaging (fMRI) and stimulus analyses, and pretty publication quality plotting in a unified and relatively rigid interface. Unlike *PsychoPy*, *PyMVPA*, *pandas*, or *matplotlib* that are very flexible and support multiple options to suit everyone's needs, the underlying philosophy of *psychopy_ext* is to act as the glue at a higher level of operation by choosing reasonable defaults for these packages and providing patterns for common tasks with a minimal user intervention. More specifically, it provides extensive and well-structured wrappers to these packages such that interaction between them becomes seamless.

## Design

### Philosophy

The overarching philosophical stance taken in *psychopy_ext* can be summarized in the following manner: *Tools must act clever*. This statement implies several design choices for a software package:

1. *Reasonable defaults.* When a package is designed with the idea that it must act clever, reasonable expectations from an end user can be matched. Unfortunately, many packages lack this quality. For example, while *matplotlib* excels in producing plots, by default it lacks publication-quality polish which is a reasonable expectation from a user.
2. *Minimal user intervention (top-down principle).* A package should be capable of producing a working end product with little effort on a user's part. Importantly, various components in the workflow should be aware of each other and able to transfer information.
3. *Intuitive interface.* A user should not struggle to grasp how to perform a certain task. Rather, as explained in PEP 20^3^, “There should be one—and preferably only one—obvious way to do it.”
4. *Encourage good habits.* In Python, code layout is not left up to a user—it is part of language specification, resulting in inherently highly readable code as compared to other programming languages. Similarly, I maintain that software should be clever enough to encourage or even require using such habits *by design*.

### Implementation

**Figure 1:**
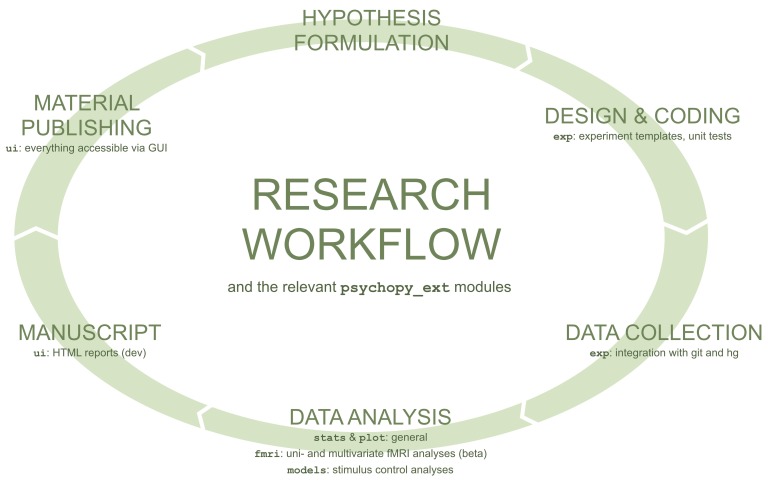
**A typical research workflow in neuroscience and psychology**. For each task, modules from *psychopy_ext* that streamline the particular task are listed. Figure adapted from Kubilius (2013).

The aim of *psychopy_ext* is to streamline a typical workflow in psychology and neuroscience research that is depicted in Figure 1. In particular, an ideal tool should:

1. Streamline as many workflow steps as possible (“be clever”).
2. Seamlessly tie together these workflow steps.
3. Facilitate reproducibility of the entire workflow.

To reach this goal, *psychopy_ext* aims to abstract common routines encountered in a typical research cycle, and wrap relevant existing packages in the format that makes them easily available to an end-user. By adhering to the design philosophy explained above, the goal is to anticipate common user's needs and provide a “magic” (or “all taken care of”) experience. This goal is achieved by employing several means.

First of all, *psychopy_ext* makes many choices for a user. For example, while there are many formats to store data collected during an experiment, only several of them facilitate sharing (White et al., 2013). Thus, unlike many other packages, *psychopy_ext* imposes that data is saved solely to a comma-delimited.csv file in the long format, which is versatile and widely adopted, and it does not support exporting to tab-delimited or Microsoft Excel's xsl/xslx files which can be potentially problematic (White et al., 2013). Such consistency in data output structure both improves project organization and significantly simplifies functions that use this data.

Moreover, *psychopy_ext* has a large number of built-in functions that ensure that an experiment or an analysis can be up and running with minimal effort on the user part. Very few things have to be specified by a user to generate working experiments, control stimuli, or produce nice looking plots. Importantly, unit testing and version control are built-in features of *psychopy_ext*, gently encouraging a user to embrace good programming practices. Similarly, access to simple image processing models is provided, allowing researchers to quickly rule out potential confounds in their stimuli prior to conducting a study and resulting in better controlled research paradigms.

Finally, *psychopy_ext* strives to integrate well with Python in order to improve coding habits. In my experience, experiments are often understood and coded as a sequence of commands. However, this intuitive model quickly breaks when more sophisticated routines and reuse of parts of code are necessary, resulting in a poor codebase organization overall. Therefore, in *psychopy_ext* experiments and analyses are defined as classes with their methods intended for a single task only. Such design subjects users to learn and actively benefit from object-oriented programming (OOP) and modularity. Moreover, the code automatically becomes more readable.

While adopting a particular workflow might induce a steep learning curve, I maintain that common templates facilitate code clarity, comprehension, and reproducibility (Wilson et al., 2012). In fact, multiple automations featured in *psychopy_ext* are solely possible due to this rigid structure. On the other hand, introducing such templates does not impede flexibility because in the OOP approach a user is free to customize everything to her own needs.

### Technical details

#### Scope and audience

*Psychopy_ext* is a Python package that wraps together other Python tools for streamlining research, including *PsychoPy*, *pandas*, *matplotlib*, and *pymvpa2*. As such, it is not a standalone tool; rather a user is expected to have Python with relevant dependencies installed (which is the case for *PsychoPy* users that *psychopy_ext* is directly targeted to). Moreover, users are expected to be at least somewhat familiar with OOP as *psychopy_ext* takes an extensive advantage of it.

#### Dependencies

*Psychopy_ext* depends on *PsychoPy*^4^ (version 1.70+) and *pandas*^6^ (version 0.12+), both of which are provided by the Standalone *PsychoPy* distribution. To benefit from automatic docstring conversion to instruction displays during experiments, *docutils*^6^ is required. *Seaborn*^7^ (version 0.1+) is also highly recommended for extremely beautiful plots (otherwise it defaults to good-enough *pandas* parameters). For fMRI analyses, *pymvpa2*^8^ (version 2.0+) and *nibabel*^9^ are required.

#### Installation

*Psychopy_ext* is part of the Standalone PsychoPy distribution. Inexperienced users are encouraged to obtain it by downloading this distribution because it comes packaged with *psychopy_ext* dependencies as well as a number of other scientific packages. More advanced users can install *psychopy_ext* using the standard *pip* installation procedure (*pip install psychopy_ext*) provided they have dependencies already installed. However, for maximal flexibility users are encouraged to download the source package of *psychopy_ext* and place it together their experiment projects without ever installing it.

#### Documentation

*Psychopy_ext* provides an extensive user manual and a growing list of demos, including behavioral and fMRI experiments, single and multiple task experiments, and fixed length and adaptive (staircase) paradigms.

#### Creating your own project

The easiest way to get started on *psychopy_ext* is to copy the entire *demos* folder, choose a demo most closely resembling user's paradigm, and adjust it accordingly.

#### License

*Psychopy_ext* is distributed under GNU General Public License v3 or later^10^.

#### Stability

*Psychopy_ext* has been 4 years in development and has reached a stable core architecture with the current release of version 0.5. It is included in the Standalone PsychoPy distribution since version 1.79. All modules in the package except for *fmri* (which is provided as a *beta* version) are automatically tested with unit tests.

## Overview of currently available tools

Below I evaluate currently available tools using these criteria and highlight where *psychopy_ext* could be used to provide a better user experience in the context of psychology and neuroscience.

### Streamlining within package

Most currently available tools for researchers excel at providing building blocks for specific tasks but typically lack standard routines (or templates) to easily integrate these blocks together. For example, creating a Gabor stimulus in *PsychoPy* is simple and achieved by calling a single command. However, a real experiment is never limited to a mere presentation of a stimulus but rather consists of a series of manipulations on these primitive building blocks. Crucially, however, many of these manipulations are not pre-defined in *PsychoPy*. For instance, instructions are usually shown at the beginning of the experiment, trials consist of showing several stimuli in a row (e.g., fixation, stimulus, fixation, and recoding participant's response), data and runtime logs are recorded to data files, yet none of these steps have the same single command access as the Gabor patch.

Presumably, such limitation is not a shortcoming but rather the wide-spread philosophy that each experiment might require a different approach and a user should be free to combine building blocks for a particular task at hand. However, as illustrated above, upon imposing certain assumptions even complex workflows can often be abstracted and thus streamlined to a large extent, in effect requiring only minimal customization on the user part.

Many other packages used by researchers suffer from a similar limitation. For example, while *matplotlib* can quickly produce plots, with default settings they are rather unappealing and a lot of handiwork is required each time to prepare figures for publication. It is possible that publishable quality is not the major goal of *matplotlib* or, similarly to *PsychoPy*, requirements for figures might be thought to vary case-by-case. However, as *seaborn* successfully demonstrates, pretty publication quality plots can be made even for complex analyses by default, and it is therefore incorporated in *psychopy_ext*.

### Integration across packages

Most currently available tools for researchers address only a single step of the typical workflow depicted in Figure 1. For example, *PyMVPA* and *pandas* are powerful libraries for data analysis but they make little or no assumptions how data was obtained and what its structure could be. Lack of such assumptions make these tools very flexible and applicable to nearly all contexts but, unfortunately, at a cost of users having to connect separate workflow steps manually.

Consider, for example, *pandas'* generic Split-Apply-Combine routine which allows users to split data into groups according to a certain criterion, then apply a function to each of those groups, and combine the results into a new data structure. Such routine is clearly useful for data analysis in general. However, many psychologists will end up using this routine to compute average response time or accuracy among participants. With the existing Split-Apply-Combine routine it would be somewhat tedious to implement this computation, but given the ubiquity of it a researcher can rightfully expect it to be available out of the box. However, *pandas* is not specialized for neuroscience and thus cannot provide such function. Similarly, *PsychoPy*, the leading Python package for designing and coding experiments, currently does not provide an interface for conducting data analysis either.

To the best of my knowledge, there are no tools currently that would directly connect experiments, analyses, and simulations. However, there have been several attempts to better integrate research workflow. One notable effort in neuroscience community is the *NeuroDebian* project (Halchenko and Hanke, 2012) that provides a platform with many tools used by neuroscientists available with a single installation command. Since the entire operating system and packages can be wrapped in a Virtual Machine, this project provides a viable solution to a difficult problem of sharing the entire research workflow in such a way that anybody would be guaranteed to be able to run the project.

Alternative solutions include research-oriented workflow management systems such as VisTrails^11^, Taverna^12^, Galaxy^13^, and ActivePapers^14^ that link separate workflow components together into one. These systems are very powerful and versatile yet might be too elaborate for the typically modest workflows that neuroscientists and psychologists share. Moreover, a user nonetheless has to implement many types of communication between nodes in the workflow manually.

There are also a number of tools that integrate analysis output with manuscript production. Most notably, *Sweave* and *knitr* are popular packages for dynamic report generation that enable embedding R code outputs into text documents (see Open Science Paper^15^ and Wallis et al.^16^, 2014, for examples of usage in research). A similar *Python* implementation of *Sweave* is available via *Pweave*. There are also a number of alternatives for incorporating text into Python code, such as IPython Notebook (Perez and Granger, 2007), *pylit*^17^, *pyreport*^18^, or for incorporating Python code into LaTeX documents (*pythonTeX*^19^), as well as language-independent solutions like *dexy.it*^20^. However, it is not clear at the moment which of these approaches will be adopted by the community at large, but in the future one of these packages could also be integrated in the *psychopy_ext* framework.

### Reproducibility

Research output should be completely reproducible. However, in practice, this is often not the case. Researchers often fail to organize their code base and analyses outputs, do not keep track of changes, neglect to comment code, and usually complete a number of steps in their workflow manually, which make an exact reproduction of output hardly possible even for the original author. Unfortunately, few efforts have been put forward to address these issues.

One simple way to improve reproducibility is provided by version control systems such as *git* or Mercurial (*hg*). These systems document changes in code, potentially allowing going back and inspecting parameters that produced a particular output. A similar but somewhat more focused towards research approach is implemented in the *Sumatra* package (Davison, 2012). *Sumatra* is meant for keeping records of parameters in projects based on numerical simulations. It keeps a record of parameters used at each execution, and also allows providing comments about simulations, link to data files and so on. Both version control systems and *Sumatra* can significantly increase organization and transparency. However, due to their relative complexity and a requirement of a special commitment from a researcher to maintain a log of activity, such systems are not widely adopted by researchers in the field. Arguably, such tools would work best if they were implemented to work implicitly, which is the approach that *psychopy_ext* enacts. Moreover, reproducibility is usually poor due to lack of instructions how to reproduce given results and what parameters should be used rather than because of a mere lack of code history. An ideal tool should therefore encourage code documentation and overall organization.

### Conclusion

Overall, a number of excellent specialized Python packages are available to researchers today yet there does not appear to be a package that would match the three criteria I proposed for an “ideal” tool. Current tools largely do not provide a top-down approach to a typical scientific routine. In particular, the entire workflow should be possible to run largely automatically with only an occasional user intervention where customization to particular needs (such as defining stimuli or selecting analysis conditions) is necessary.

## *Psychopy_Ext* components

### Overview

*Psychopy_ext* is composed of six largely distinct modules: user interface (extends *argparse* and *psychopy.gui*), experiment creation (extends *PsychoPy*), (generic) data analysis (extends *pandas*), fMRI data analysis (extends *pymvpa2*), modeling and plotting (extends *matplotlib*). The modules easily combine together in order to streamline user's workflow (Figure 1).

### Project structure

**Figure 2:**
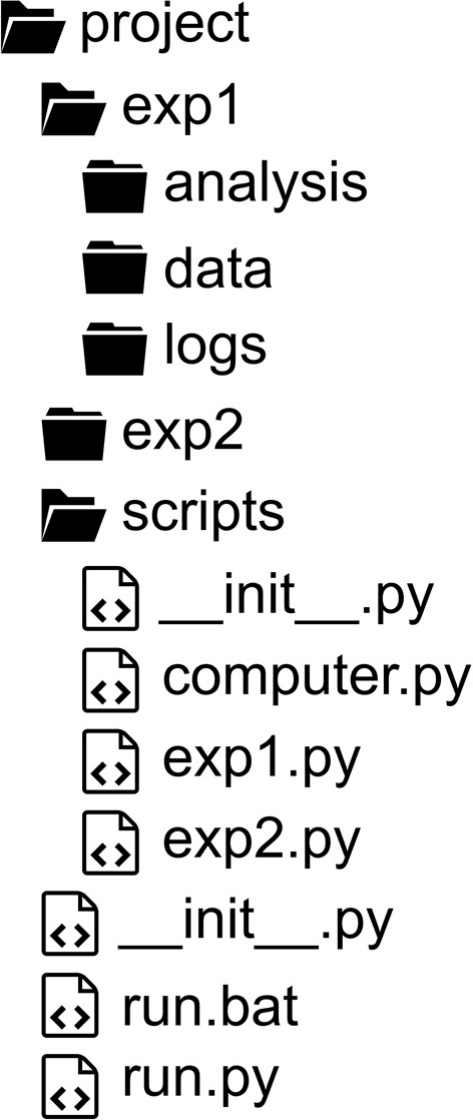
**A recommended project structure**.

*Psychopy_ext* assumes the position that all project-related materials must reside together, organized in a rigid and consistent folder and file naming structure (Figure 2). Data and other output files are stored in separate folders for each study, all of which reside in the Project folder (unless specified otherwise). Such organization already improves researcher's habits with no extra effort and significantly facilitates collaboration and reproducibility.

**Figure 3:**
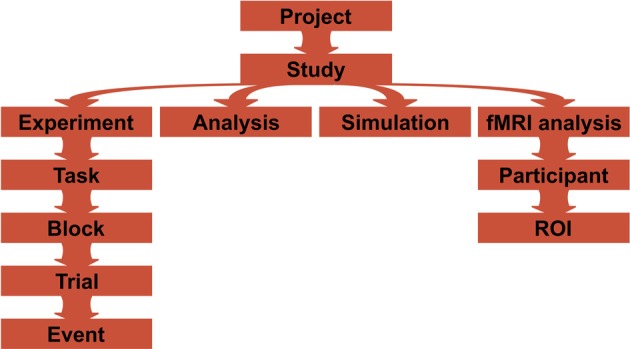
**A typical project structure**. A project is composed of one or more studies that are defined in separate scripts, and each of them can have experiment, analysis, simulation, or fMRI analysis classes defined. Experiments can have one or more tasks (like a training paradigm and then testing performance), that can be further subdivided into smaller blocks, providing short pauses in between the blocks. Each block has a list of trials that are composed of a list of events. For fMRI analyses, computations occur per participant per ROI.

A Project is assumed to consist of multiple Studies, each defined by a separate Python script (Figure 3). A Study consists of experiment, analysis, simulation, and any other user defined classes. Any non-private methods defined by these classes (such as running the experiment, displaying all stimuli, plotting average response times and so on) can be called via GUI or a command-line interface (see *User interfaces*). It is also possible to limit callable methods by providing *actions* keyword to the class constructor.

All these scripts are not meant to be called directly. Rather, a single file, *run.py*, is used in order to provide a unified interface. Running this file will open a GUI where a user can choose which study to run and what parameters to use, or parameters can be passed directly via a command-line interface. Finally, parameters specific to particular setups, such as monitor sizes and distances, can be specified in *computer.py* file, providing a seamless functioning across multiple machines.

### User interfaces

**Figure 4:**
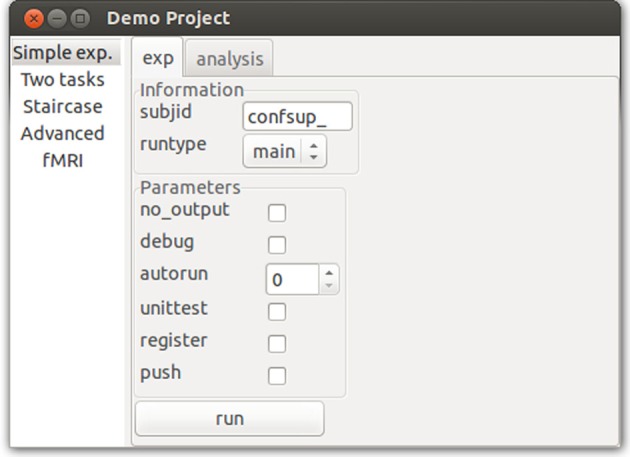
**Graphical user interface (GUI)**. *Psychopy_ext* converts *info* and *rp* parameters found in the class definition of an experiment or an analysis into GUI widgets, and methods into buttons. Note that this GUI is completely automatically generated from a class definition and does not require user intervention.

To facilitate the goal of unifying all research steps, *psychopy_exp* module *ui* automatically generates command-line (CLI) and graphic user interfaces (GUI) from user's code. It scrapes through all scripts (using Python's *inspect* module) seeking for non-private classes and functions, and extracts initialization parameter values stored in a *name*, *info* and *rp* variables. A *name* is used as an alias in CLI to call that class. *info* is a concept inherited from PsychoPy's *extraInfo* and is defined as a dictionary of *(key, value)* pairs of information that a user wants to later save in an output file (e.g., participant ID). Finally, *rp* define parameters that will not be saved in the output but control how a script runs. For example, they could control whether output files are saved or not, whether unit tests should be performed and so on. A number of standard *rp* options are already built-in (see Figure 4).

When *run.py* file is called, a GUI is generated using these parameters (Figure 4). A GUI is a *wxPython* app where different studies are presented in a *wx.Listbook*, and each task (running the experiment or performing analyses) is nested in its tabs as a *wx.Notebook* with input fields generated from *info* and *rp* (note different widgets for different input types) and buttons created for available actions (e.g., plot response time data or plot accuracy). As such, *psychopy_ext* significantly extends *PsychoPy's* functionality where only a simple dialog box is available via its *gui* module.

Most users will benefit from the automatically generated GUI for a number of reasons. First, running and rerunning experiments or analyses while manipulating various parameters becomes much easier, merely a matter of ticking the right boxes and clicking buttons rather than editing source code every time. Moreover, when a button is pressed, a new subprocess is initiated to run a particular task. Thus, a user can keep the GUI open and continue changing and rerunning the code with the same parameters, which greatly speeds up development. Finally, rerunning the project becomes much easier for other researchers.

Some users will also appreciate a powerful CLI for running tasks. CLI allows users to call required tasks directly without the intermediate GUI step. It uses syntax comparable to Python's *argparse* with a difference that positional arguments (script, class and function names) are before optional arguments, for example (also see Figure 3):

python run.py main exp run ––subjid
              subj_01 ––no_output



If no arguments are provided (i.e., python run.py), a GUI is generated instead.

Note that using Python's default *argparse* would be considerably less convenient as one would have to manually update *argparse* definitions every time a new option or function is introduced to a class.

Moreover, it is important to understand that such user interfaces would not otherwise be possible if a particular code structure were not imposed by *psychopy_ext*. In order to be able to use an interface, a user is forced to organized her code into classes and functions, and immediately choose which parameters can be manipulated by a user. Such organization brings significant clarity to the code (variables are not scattered around the code) and teaches a user the benefits of OOP. Moreover, reproducibility is inherently built in the code and does not require any special preparation before publishing. In fact, the significant time investment in preparing code for public is often cited as one of the reasons researchers do not publish their code by default (Barnes, 2010), thus *psychopy_ext* might help to alter this tendency.

### Running experiments

**Figure 5:**
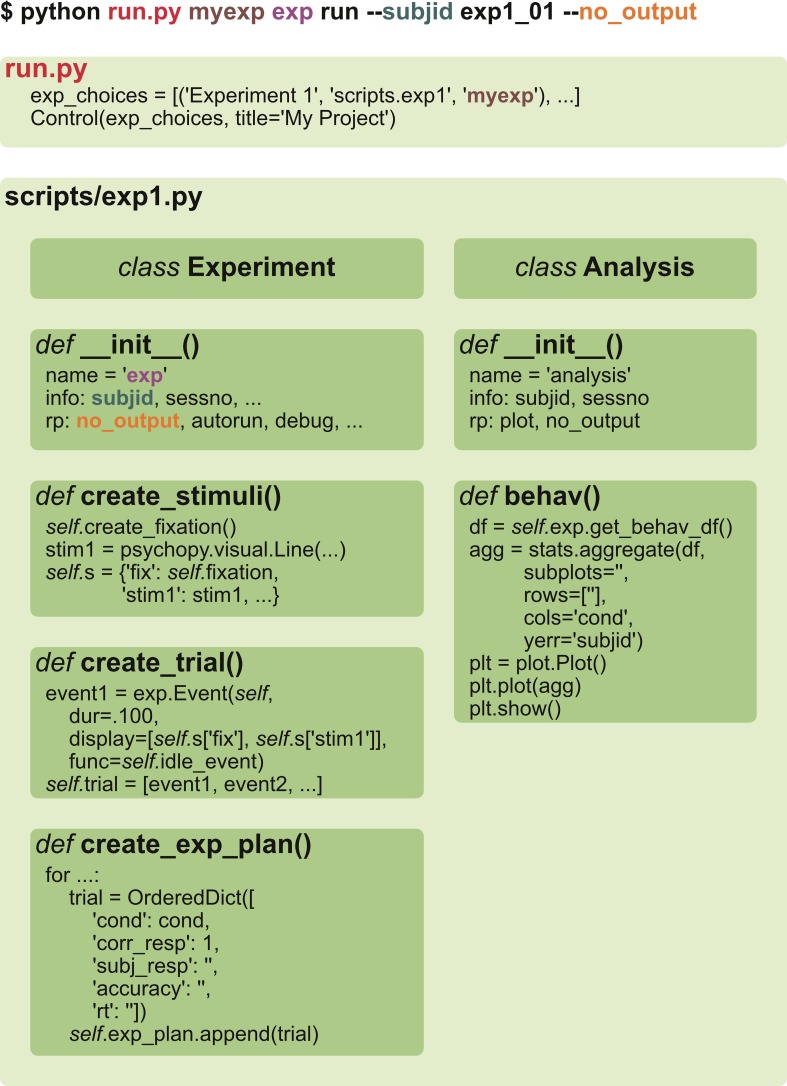
**A typical experiment and analysis structure**. A user executes *run.py* file either without any arguments (resulting in a GUI) or with them (as shown in this example). Then, relevant scripts (brown), classes (purple) and methods (black) are found and performed. A minimal structure of the script is depicted in the lower panel. The user only has to specify stimuli, trial structure, and the list of trials for experiment, and an analysis method for analysis.

The experiment module *exp* provides a basic template and multiple common functions for presenting stimuli on a screen and collecting responses. An experiment is created by defining a class that inherits from the *Experiment* class, thus gently introducing the concept of inheritance. This may be somewhat unusual to many users used to linear experimental scripts but the advantage is that a number of functions can readily be used from the parent class or overridden if necessary. Only stimulus definition, trial structure, and trial list creation are always defined in the child class (see Figure 5; Listing 1). Again, a good practice of modularity becomes natural in this setting.

**Listing 1:**
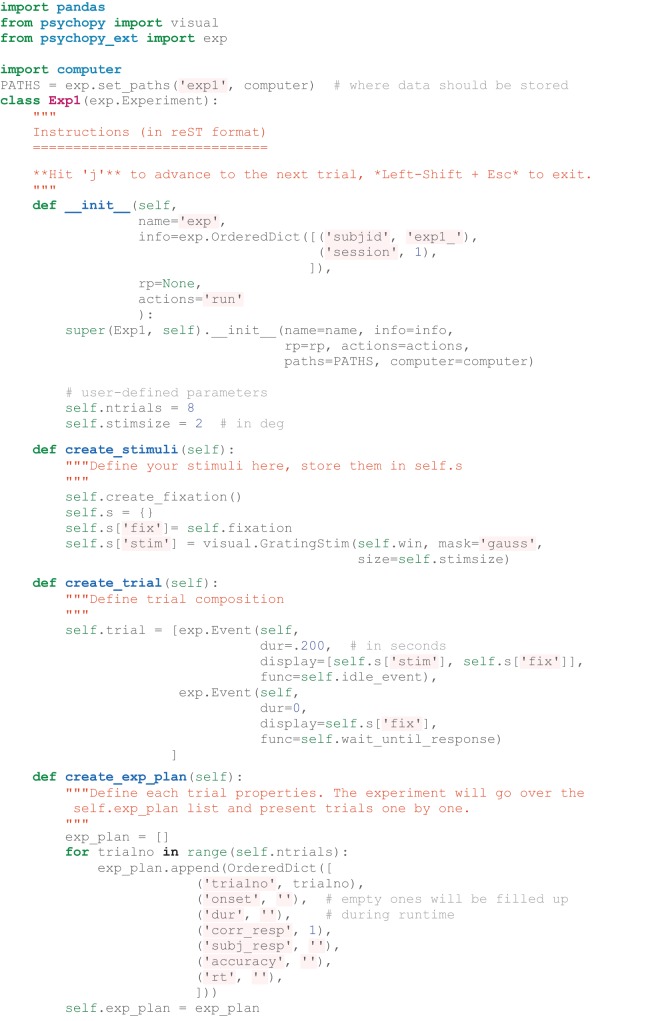
**The simplest fully functional experiment (with data and log files generated) that shows eight trials of Gabor grating and waits for response in between them**.

Listing 1 shows how to create a simple experiment in *psychopy_ext* consisting of a single task only. To have more than one task (e.g., first training on a particular set of stimuli and then testing performance), multiple Task classes (inheriting from the *exp.Task* class) can be defined separately with the same basic structure as demonstrated above (see Figure 3). The tasks should be stored in a self.tasks variable in the main Experiment class, which would then call each task one by one during the runtime. Each Task can further be divided into Blocks with short pauses in between by defining a self.blockcol variable that refers to a particular column in self.exp_plan variable where block number is stored. Blocks consist of Trials that consist of Events (e.g., show a fixation, show a stimulus, show a fixation and wait for response). The flow of these components is handled by the *exp* module; a user is required to only define these structures (though a deeper customization is a matter of overriding the default methods, of course). Experiment, Task, and Block classes have before and after methods that allow to customize what happens just before and right after each of them are executed. These methods are typically useful to define instruction or feedback displays between tasks.

Beyond streamlining experiment creation, the Experiment and Task classes offer several methods to address typical researcher needs. First, every experiment inherited from these classes has a built-in automatic running functionality which allows users to quickly go through the entire experiment, in the essence acting as unit testing. Moreover, keyboard input is simulated such that responses could be collected and analyzed. A user can even define simulated responses such that they would match the expected outcome of the experiment. Such manipulation is especially handy when a novel analysis technique is used and the user is not confident that it was implemented correctly. Together, this function enables users to quickly verify that both experimental and analysis code are working properly prior to collecting any data.

The Experiment class also simplifies study registration and data collection processes by integrating with version control systems *git* and Mercurial (*hg*). If an appropriate flag is selected, at the end of experiment new data and log files are committed and pushed to a remote repository. Therefore, this feature allows an automatic data sharing among collaborators, creates an instant backup, and prevents users from tampering with raw data.

### Data analysis and plotting

**Figure 6:**
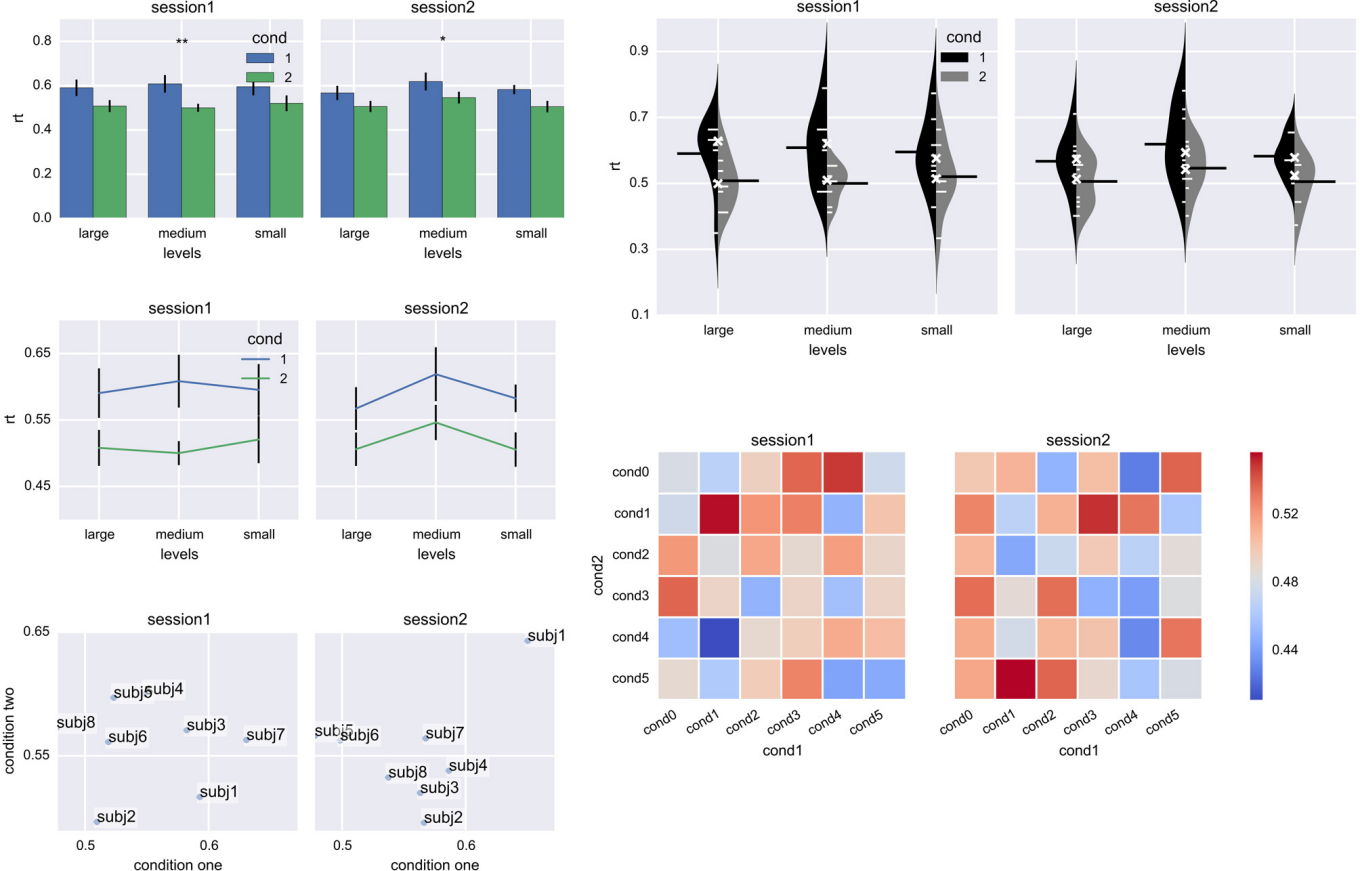
**Plots generated by the *plot* module. Left panel:** bar plot, line plot, and scatter plot; **right panel**: bean plot (Kampstra, 2008) and matrix plot. The pretty color scheme is applied by default and subplot layout, tick spacing, labels and other plot properties are inferred from the original data without any manual input. *p < 0.05, **p < 0.01.


Data analysis (*stats*) and plotting (*plot*) modules aim to simplify basic data analysis and plotting. The *stats* module tailors *pandas* functionality for typical analysis patterns in neuroscience and psychological research. In particular, it provides the *aggregate* function which splits data into groups according to a certain criterion (e.g., a participant ID) and applies a requested function to each group (an average, by default), returning a *pandas.DataFrame*. For example, to aggregate response times for each participant separately and then plot averaged data in two subplots (per session) with three levels on the x-axis and two conditions in different colors with error bars (Figure 6, bar plot), the following command is used:

agg = stats.aggregate(df, rows='levels',
      subplots='subplots', cols='cond',
      yerr='subjID', values='rt')


This results in a DataFrame with subplot, level, and condition labels its index, and an average per participant (as specified by yerr keyword) in columns.

The agg variable can be directly used for plotting, vastly simplifying and improving plotting experience:

plt = plot.Plot()
agg = plt.plot(agg, kind='bar')
plt.show()



On top of plotting data, the *plot* function also:

- creates the required number of subplots
- formats and labels axes
- formats legend
- draws error bars
- for line and bar plots, performs a *t*-test (either one-sample or two-samples) and displays results with one or more stars above
- chooses pretty color and layout options reminiscent of R's *ggplot2* using *seaborn*, or *pandas* default color scheme if *seaborn* is not available.

Observe that the resulting plot is immediately correctly formatted because the *aggregate* function recorded data layout information in the index and column names. Moreover, in many cases it has enough information (labels, error bars) and polish for publication, in part thanks to *seaborn* package. (In future releases, a tighter integration with *seaborn* is planned.)

Also note that plotting module takes a slightly different approach from *matplotlib* by requiring to initialize the Plot() class for each plot. Due to this change, it becomes possible to easily and automatically create figures with multiple subplots. For example, a subplot does not need to be created prior to plotting; it is automatically created upon the next call of the *plot* function.

### Functional magnetic imaging (fMRI) analysis

Preprocessing of functional magnetic imaging (fMRI) data has become mainstream as a result of a number of robust and free analysis tools, such as *SPM* (Ashburner and Friston, 2005), *FreeSurfer*^21^, or *AFNI* (Cox, 1996). More recently, multivariate pattern analysis (MVPA) has become available to many researchers thanks to packages such as *PyMVPA* (Hanke et al., 2009). However, similar to stimulus presentation packages, many free fMRI analysis tools lack standard “plug-and-play” routines that would allow users to carry out data analysis automatically. For example, setting up a generic routine in *PyMVPA* that would go over all participants, extract relevant regions of interest (ROIs), perform and plot correlational or support vector machine (SVM) analysis is not possible because researchers usually have their own preferred workflows.

However, in *psychopy_ext* this goal becomes viable due to a well-controlled data structure. The *fmri* module consists of the *Preproc* and the *Analysis* classes that only require relevant participant ID's and ROIs to be specified to carry out analyses in full. The *Preproc* class generates batch scripts to compute beta- or *t*-values using Statistical Parametric Mapping toolbox (Ashburner and Friston, 2005). In future releases, this functionality could be extended to automate the entire preprocessing workflow using *Nipype* (Gorgolewski et al., 2011) or *Lyman*^22^ packages. The *Analysis* class uses preprocessed data to display regions of interest, plot changes in the fMRI signal intensity and perform univariate (BOLD signal averages for each condition) and multivariate (MVPA) analyses (however, group analyses are not implemented).

For MVPA analyses, two most popular analysis approaches, namely, correlational and SVM analyses, are provided. Both are implemented in a similar fashion. First, data is normalized for each run by subtracting the mean across conditions per voxel (for correlational analyses) or across voxels per condition (for SVM analyses; Kubilius et al., 2011). Next, data is divided in two halves (for correlations) or into roughly 75% of training data (to train the SVM) and 25% of test data (to test the SVM performance). Pair-wise correlations or pair-wise decoding for all possible combinations are then computed. For SVM, by default a linear nu-SVM kernel is used and an average of the test set is taken to improve the performance (Kubilius et al., 2011). In order to achieve a more stable performance, this computation is performed for 100 iterations by randomly choosing the splits of samples. Outputs of these computations are provided in a standard *pandas* DataFrame format, which can further be used to plot the results within the same *psychopy_ext* framework.

Although this module is experimental at the moment due to the lack of relevant unit tests, it has already been used in several published or submitted papers (Kubilius et al., 2011; Kubilius et al., unpublished results). Moreover, a user can easily adapt a particular analysis details to her liking while still benefiting from the implementation of the global routine.

### Simulations

In many vision experiments it is important to verify that the observed effects are not a mere result of some low-level image properties that are not related to the investigated effect. Several simple models have been used in the literature to rule out such alternative explanations, including computing pixel-wise differences between conditions (e.g., Op de Beeck et al., 2001), applying a simple model of V1 such as the GaborJet model (Lades et al., 1993), or applying a more complex model of the visual system such as HMAX (Riesenhuber and Poggio, 1999). *Psychopy_ext* provides a wrapper to these models so that they could be accessed with the same syntax, namely, by passing filenames or *numpy* arrays of the images that should be analyzed and compared:

model = models.HMAX()
model.compare(filenames)



To get raw model output, the *run* command can be used:

model = models.HMAX()
out = model.run(test_ims=test_fnames,
                train_ims=train_fnames)



Pixel-wise differences model is the simplest model for estimating differences between images. Images are converted to grayscale, and a Euclidean distance is computed between all pairs of stimuli, resulting in an n-by-n dissimilarity matrix for *n* input images (Op de Beeck et al., 2001).

GaborJet model (Lades et al., 1993) belongs to the family of minimal V1-like models where image decomposition is performed by convolving an image with Gabor filters of different orientation and spatial frequency. In the GaborJet model, convolution is performed using 8 orientations (in the steps of 45°) and 5 spatial frequencies on a 10-by-10 grid in the Fourier domain. The output consists of the magnitude and phase of this convolution (arrays of 4000 elements), and the sampled grid positions. For comparing model outputs, only magnitudes are usually used to compute an angular distance between the two output vectors (Xu et al., 2009). In *psychopy_ext*, the code has been implemented in Python by following the MATLAB implementation available on Irving Biederman's website^23^.

HMAX model (Riesenhuber and Poggio, 1999) has been proposed as a generic architecture of the visual cortex. It consists of four image processing layers and an output layer. Initially, a convolution between the image and Gabor filters of four orientations (in the steps of 45°) and 12 spatial frequencies (range: 7–29 px) grouped into four channels is computed (layer S1). Next, a maximum of outputs of the same orientation over each spatial frequency channel is taken (layer C1). Outputs of this operation are pooled together in 256 distinct four-orientation configurations (for each scale; layer S2), and a final maximum across the four scales is computed (layer C2), resulting in an output vector with 256 elements. If training data is provided, these responses can further be compared to the stored representations at the final view-tuned units (VTU) layer. In *psychopy_ext*, the code has been implemented in Python by following the MATLAB implementation by Minjoon Kouh and the original implementation available on Max Riesenhuber's website^24^. (Note that the current implementation of HMAX as provided by Poggio lab is much more advanced than the one implemented in *psychopy_ext*.)

## Limitations

*Psychopy_ext* debuted publically in November 2013 and thus has not been adopted and extensively tested by the community yet. It is therefore difficult to predict the learning curve of the underlying *psychopy_ext* philosophy and to what extent it resonates with the needs of the community. For example, many researchers are used to linear experimental and analysis scripts, while *psychopy_ext* relies on object-based programming concepts such as classes and modular functions in order to provide inheritance and flexibility. However, object-oriented approach also means that whenever necessary functions are not available directly from *psychopy_ext* or do not meet user's needs, they can be overridden or used directly from the packages that are extended, often (but not always) without affecting the rest of the workflow.

Furthermore, *psychopy_ext* was designed to improve a workflow of a typical *PsychoPy* user. Researchers that use other stimulus generation packages or even different programming languages (such as *R* for data analyses) will not be able to benefit from *psychopy_ext* as easily. Such limitation is partially a design choice to provide workflows that depend on as few tools as possible. Python has a large number of powerful packages and *psychopy_ext* is committed to promoting them in favor of equivalent solutions in other languages. Nonetheless, when an alternative does not exist, users can easily interact with their *R* (via rpy2^25^), *C/C++* (via Python's own *ctypes*), MATLAB (via pymatlab^26^ or mlab^27^) and a number of other kinds of scripts.

## Discussion and future roadmap

Four years into development, *psychopy_ext* is already successfully addressing a number of issues encountered in streamlining a typical research workflow and its reproducibility. By design, it enables researchers to produce well-organized projects with a number of typical steps automated, providing prebaked templates and interfaces for common tasks, and implementing default unit testing in a form of customizable simulations. These projects can be rapidly developed as *psychopy_ext* requires only a minimal customization by a user to run and are easily reproducible via an automatically generated GUI.

In future releases *psychopy_ext* will introduce more tools to streamline typical routines encountered by psychologists and neuroscientists. Beyond small improvements, there are several intriguing possibilities that *psychopy_ext* could explore.

To begin, an interesting and, arguably, quite intuitive approach to reproducibility has been recently introduced by Stevens et al. (2013) in their Python package called *Lancet*. Often, reproducibility is understood as a *post-hoc* feature where a researcher cleans up and organizes her code just prior to publication. Since this final code has a very different structure from a naturally exploratory format of code in day-to-day research, extra effort is required from a researcher to prepare it. In contrast, *Lancet* allows exploratory research to naturally grow from IPython Notebooks into more complex workflows where external processes can be launched and tracked from the same pipeline. Such natural code evolution is also encouraged in *psychopy_ext* but instead by defining new classes and functions for new branches of exploration. Introducing functionality of both approaches might be fruitful to explore in future releases of *psychopy_ext*.

Furthermore, given a neat integration of experimental and analysis workflow it would be possible to automatically produce reports of the experimental and analyses parameters and outputs. Upon integration of experiment's parameters, this feature could even lead to an initial draft of a manuscript with Methods and Results sections already partially prefilled. In fact, in the development branch of *psychopy_ext*, a very primitive approach to generating reports of analyses in a single HTML file is already available. More robust results could be achieved by integrating one of the Python packages for combining text and code as mentioned in the *Integration* section.

Integration of resources could be further fostered by a general project management tool. This tool could provide access to all project materials, as well as track and display changes in them, similar to *Projects*^28^ software for Mac OS or a number of open and platform-independent workflow systems mentioned in the *Integration* section, especially VisTrails since it is Python-based. Alternatively, such tool could be browser-based, thus enabling researchers to access their projects from anywhere, and it could integrate well with the existing browser-based solutions, such as data plotting libraries.

Moving toward more GUI-based solutions also opens a possibility to improve user experience in designing an experiment and analysis. For example, experiment creation in *psychopy_ext* is already semantically structured: projects consist of experiments that consist of tasks that consist of blocks, trials and events. Such organization easily maps onto a GUI with blocks representing different components, somewhat akin to *PsychoPy Builder*. Similarly, a pivot table or pivot chart option, reminiscent of the one in *Microsoft Excel*, could be provided to allow a quick exploration of data.

Taken together, *psychopy_ext* provides a transparent and extendable framework for developing, sharing and reusing code in neuroscience and psychology.

### Conflict of interest statement

The author declares that the research was conducted in the absence of any commercial or financial relationships that could be construed as a potential conflict of interest.

## Footnotes

^1^http://pandas.pydata.org

^2^http://statsmodels.sourceforge.net

^3^http://www.python.org/dev/peps/pep-0020/

^4^http://www.psychopy.org/

^5^http://pandas.pydata.org/

^6^http://docutils.sourceforge.net/

^7^http://stanford.edu/~mwaskom/software/seaborn

^8^http://www.pymvpa.org/

^9^http://nipy.org/nibabel/

^10^http://www.gnu.org/licenses/

^11^http://www.vistrails.org

^12^http://www.taverna.org.uk/

^13^http://galaxyproject.org/

^14^https://bitbucket.org/khinsen/active_papers_py

^15^https://github.com/cpfaff/Open-Science-Paper

^16^https://github.com/tomwallis/microperimetry_faces

^17^http://pylit.berlios.de/

^18^http://gael-varoquaux.info/computers/pyreport/

^19^https://github.com/gpoore/pythontex

^20^http://www.dexy.it/

^21^http://surfer.nmr.mgh.harvard.edu/

^22^http://stanford.edu/~mwaskom/software/lyman/

^23^http://geon.usc.edu/GWTgrid_simple.m

^24^http://riesenhuberlab.neuro.georgetown.edu/hmax/index.html

^25^http://rpy.sourceforge.net/rpy2.html

^26^http://molflow.com/pymatlab.html

^27^https://github.com/ewiger/mlab

^28^https://projects.ac/
